# AI at the Bedside of Psychiatry: Comparative Meta-Analysis of Imaging vs. Non-Imaging Models for Bipolar vs. Unipolar Depression

**DOI:** 10.3390/jcm15020834

**Published:** 2026-01-20

**Authors:** Andrei Daescu, Ana-Maria Cristina Daescu, Alexandru-Ioan Gaitoane, Ștefan Maxim, Silviu Alexandru Pera, Liana Dehelean

**Affiliations:** 1 Doctoral School Department, “Victor Babes” University of Medicine and Pharmacy, 300041 Timisoara, Romania; andrei.daescu@umft.ro; 2 Neurosciences Department, Discipline of Psychiatry, “Victor Babes” University of Medicine and Pharmacy, 300041 Timisoara, Romania; lianadeh@umft.ro; 3 Department of Psychiatry, Timis County Emergency Clinical Hospital “Pius Brinzeu”, 300723 Timisoara, Romania; gaitoane.alexandru-ioan@hosptm.ro (A.-I.G.); stefan.maxim@rezident.umft.ro (Ș.M.); silviu.pera@rezident.umft.ro (S.A.P.)

**Keywords:** bipolar disorder, major depressive disorder, first episode, psychiatry, artificial intelligence, machine learning, diagnostic accuracy, meta-analysis, AUC, imaging, biomarkers

## Abstract

**Background**: Differentiating bipolar disorder (BD) from unipolar major depressive disorder (MDD) at first episode is clinically consequential but challenging. Artificial intelligence/machine learning (AI/ML) may improve early diagnostic accuracy across imaging and non-imaging data sources. **Methods**: Following PRISMA 2020 and a pre-registered protocol on protocols.io, we searched PubMed, Scopus, Europe PMC, Semantic Scholar, OpenAlex, The Lens, medRxiv, ClinicalTrials.gov, and Web of Science (2014–8 October 2025). Eligible studies developed/evaluated supervised ML classifiers for BD vs. MDD at first episode and reported test-set discrimination. AUCs were meta-analyzed on the logit (GEN) scale using random effects (REML) with Hartung–Knapp adjustment and then back-transformed. Subgroup (imaging vs. non-imaging), leave-one-out (LOO), and quality sensitivity (excluding high risk of leakage) analyses were prespecified. Risk of bias used QUADAS-2 with PROBAST/AI considerations. **Results**: Of 158 records, 39 duplicates were removed and 119 records screened; 17 met qualitative criteria; and 6 had sufficient data for meta-analysis. The pooled random-effects AUC was 0.84 (95% CI 0.75–0.90), indicating above-chance discrimination, with substantial heterogeneity (I^2^ = 86.5%). Results were robust to LOO, exclusion of two high-risk-of-leakage studies (pooled AUC 0.83, 95% CI 0.72–0.90), and restriction to higher-rigor validation (AUC 0.83, 95% CI 0.69–0.92). Non-imaging models showed higher point estimates than imaging models; however, subgroup comparisons were exploratory due to the small number of studies: pooled AUC ≈ 0.90–0.92 with I^2^ = 0% vs. 0.79 with I^2^ = 64%; test for subgroup difference Q = 7.27, df = 1, *p* = 0.007. Funnel plot inspection and Egger/Begg tests found that we could not reliably assess small-study effects/publication bias due to the small number of studies. **Conclusions**: AI/ML models provide good and robust discrimination of BD vs. MDD at first episode. Non-imaging approaches are promising due to higher point estimates in the available studies and practical scalability, but prospective evaluation is needed and conclusions about modality superiority remain tentative given the small number of non-imaging studies (k = 2).

## 1. Introduction

Bipolar disorder (BD) and major depressive disorder (MDD) are common mood disorders that share overlapping depressive symptomatology but differ markedly in longitudinal course, treatment requirements, and prognosis. BD is defined by the occurrence of (hypo)manic or mixed episodes in addition to depression, and treatment typically centers on mood stabilizers and atypical antipsychotics, with cautious use of antidepressants to avoid switch or cycle acceleration. By contrast, MDD follows a predominantly depressive course and is often managed with antidepressants and psychotherapy. Misclassification at a first depressive episode is frequent and clinically consequential, delaying appropriate therapy, increasing relapse and suicide risk, and compounding functional impairment [1].

The differential diagnosis between BD and MDD at first presentation is therefore a high-stakes clinical task, particularly in first-episode settings where longitudinal markers of bipolarity are scarce. Clinical signals of latent bipolarity (e.g., early onset, psychomotor activation, atypical features, family history, and mixed symptoms) are variably present and inconsistently documented, and hypomanic episodes are frequently unrecognized. Objective tools capable of integrating multivariate patterns from routine assessments or ancillary tests could complement clinical judgment and improve early diagnostic accuracy [2].

Artificial intelligence (AI), particularly machine learning (ML), offers a data-driven approach to this problem by learning reproducible patterns that distinguish diagnostic categories and then generalizing those patterns to new patients. In supervised classification for BD vs. MDD, models ingest predictors, ranging from structured clinical and electronic medical record (EMR) variables to laboratory biomarkers, electrophysiology, speech/behavioral signals, actigraphy, and neuroimaging, and output a probability of class membership. Discrimination is commonly summarized by the area under the receiver operating characteristic curve (AUC), while clinically useful models should also demonstrate reasonable calibration (agreement between predicted probabilities and observed outcomes) and decision-relevant performance at prespecified thresholds [3].

A broad family of algorithms has been applied to psychiatric classification. Linear and penalized models (e.g., logistic regression with L1/L2 or elastic-net penalties) provide baseline performance and interpretability. Margin-based methods such as support vector machines (SVMs) handle high-dimensional feature spaces and nonlinear boundaries via kernels. Tree-based ensembles, including random forests and gradient-boosting frameworks such as XGBoost, capture interactions and complex feature effects with strong predictive accuracy and built-in regularization. Neural networks, from multilayer perceptrons to convolutional and sequence models, may exploit spatial or temporal structure in imaging, audio, or sensor data. Algorithm choice should be guided by the data domain, dimensionality, sample size, noise characteristics, and the need for transparency in clinical settings [2,4,5].

Methodological rigor in model validation is critical to ensure credible estimates of performance. Best practice includes strict separation of training, validation, and test data; performing all preprocessing steps (imputation, scaling, feature selection, and harmonization) fold-wise within training data only; and using nested cross-validation for hyperparameter tuning to prevent information leakage. When feasible, external validation on independent cohorts—ideally at the site level—provides a stronger assessment of generalizability. Beyond discrimination, reporting should include confidence intervals, class counts in the evaluation set, calibration metrics (slope, intercept, and Brier score), the rationale for decision thresholds, and, when appropriate, clinical utility via decision curve analysis [4,6].

Applied to the BD vs. MDD differential at first episode, AI/ML has shown promise across both non-imaging and imaging modalities. Non-imaging approaches leveraging EMR/clinical variables and blood-based measures are attractive due to lower cost, scalability, and reduced susceptibility to site effects, whereas neuroimaging may encode disease-related neurobiology but is sensitive to acquisition heterogeneity and harmonization challenges. The present study synthesizes the available evidence with a prespecified protocol, quantifies diagnostic accuracy on an appropriate effect scale, and evaluates robustness across sensitivity analyses to clarify the current readiness and limits of AI models for early differentiation of bipolar and unipolar depression. We therefore conducted a protocol-driven systematic review and meta-analysis to quantify overall diagnostic accuracy and compare imaging versus non-imaging performance for BD vs. MDD at first episode. Accurate differentiation between bipolar and unipolar depression at first presentation has direct implications for treatment selection, avoidance of antidepressant-induced mood switching, and the potential use of AI/ML models as adjunctive clinical decision-support tools rather than standalone diagnostic instruments. Unlike prior reviews that combine first-episode and chronic cohorts, we focused on first-episode presentations where diagnostic uncertainty is greatest and early treatment consequences are most substantial [7,8,9].

## 2. Materials and Methods

This systematic review and meta-analysis was conducted and reported in accordance with the PRISMA 2020 statement [10] and was prospectively registered on protocols.io (DOI: https:dx.doi.org/10.17504/protocols.io.4r3l21w5pg1y/v2), detailing the research question, eligibility criteria, data items, and planned analyses; any deviations from the protocol were documented.

We aimed to synthesize evidence on artificial intelligence/machine learning (AI/ML) models that differentiate bipolar disorder (BD) from unipolar major depressive disorder (MDD) at first episode, defined as the first documented mood episode at presentation, and to compare performance across imaging modalities (MRI and EEG) and non-imaging modalities (EMR/clinical data, voice/NLP, actigraphy, and blood/proteomic biomarkers).

We included original studies that (1) enrolled first-episode mood participants with clearly separable BD and MDD groups (i.e., patients experiencing a first documented mood episode classified as BD or MDD by the reference standard); (2) developed or evaluated a supervised ML classifier (e.g., SVM, random forest, XGBoost, penalized logistic regression, and CNN) for the diagnostic discrimination of BD vs. MDD; (3) used imaging or non-imaging input data; and (4) reported discrimination performance on an independent test set (primary outcome: AUC; secondary outcomes, when available: sensitivity/specificity at a stated threshold and calibration metrics). Both internal validation (hold-out or k-fold with a separate test split) and external validation were accepted. Apparent (training) performance alone was not included in the meta-analysis but, when relevant, was described qualitatively. First episode was defined as the first documented mood episode at the point of clinical presentation, without prior episodes requiring sustained treatment. Studies enrolling ‘recent-onset’ or prevalent cases (e.g., diagnosis within several years) were included only if first-episode participants were clearly separable; otherwise, they were excluded from quantitative synthesis.

For multi-class studies (e.g., including healthy controls or other diagnoses), we included results only if a separable BD vs. MDD comparison was reported or derivable. We excluded (1) schizophrenia or first-episode psychosis cohorts that did not allow a BD vs. MDD analysis; (2) prognosis, treatment response, service evaluation, or healthy control comparisons that were not focused on the BD vs. MDD discrimination task; (3) non-ML statistical analyses; (4) studies not focused on first-episode populations or not clearly separable as such; (5) reviews, editorials, protocols, and conference abstracts lacking analyzable data; and (6) reports with insufficient information for quantitative synthesis (e.g., no test-set AUC/CI/SE or class counts). Healthy control comparisons were deliberately excluded to maintain clinical relevance for the BD vs. MDD diagnostic dilemma; however, this design precludes inference about whether identified features reflect disorder-specific differentiation or nonspecific markers of psychiatric illness. Studies with unclear validation splits suggesting leakage or unstated threshold basis were retained for qualitative review only. To preserve a strict first-episode definition, studies with mixed or ‘recent onset’ cohorts not separable as first-episode-only were excluded from quantitative synthesis.

We systematically searched PubMed, Scopus, Europe PMC, Semantic Scholar, OpenAlex, The Lens, medRxiv, ClinicalTrials.gov, and Web of Science for records published between 2014 and 8 October 2025. A single Boolean strategy operationalized the target concepts and was adapted to each interface/field tag: (“bipolar disorder”) AND (“major depressive disorder” OR MDD OR unipolar) AND (“first episode” OR “first-episode”) AND (“machine learning” OR “artificial intelligence” OR “deep learning” OR “neural network” OR SVM OR “support vector” OR “random forest” OR XGBoost OR classification).

Where controlled vocabulary existed (e.g., MeSH in PubMed), subject headings were combined with title/abstract terms. In free-text/topic platforms (e.g., Scopus, Web of Science, and Europe PMC), we applied stepwise narrowing using (1) disorder terms, then (2) the first-episode qualifier, and then (3) AI/ML terms within title/abstract/topic fields. For sources with limited yield (e.g., Semantic Scholar, medRxiv, and ClinicalTrials.gov), we executed the fully combined query. No language or study design filters were applied at the search stage; preprints and trial records were included to mitigate publication bias.

**Figure 1:**
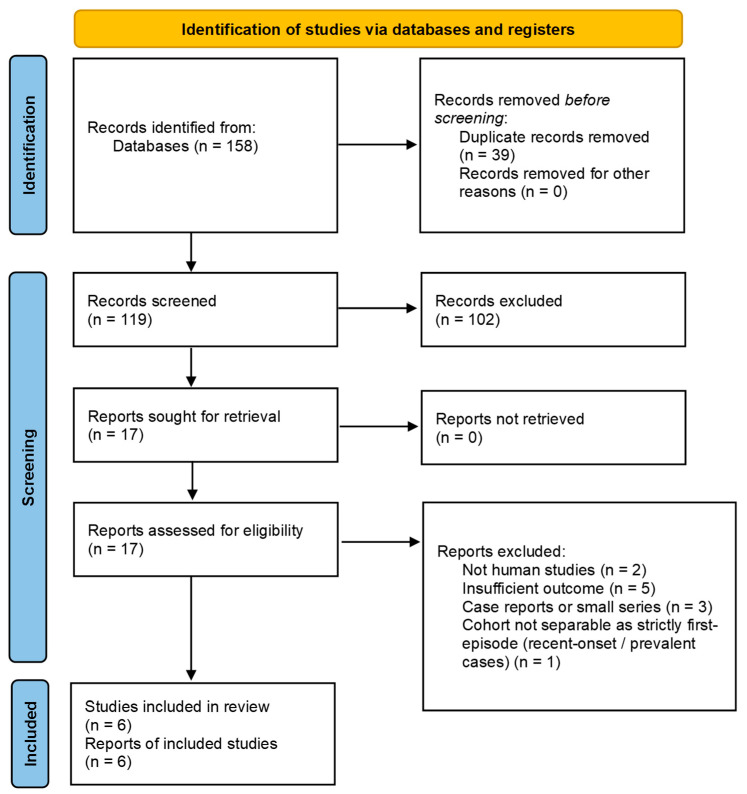
PRISMA 2020 flow diagram of study selection.

The PRISMA 2020 flow diagram (Figure 1) summarizes study selection, with the principal reasons for full-text exclusion shown beneath the relevant nodes (e.g., topic misalignment; interposed/added pathology; no AI/ML; not first-episode; wrong task; and insufficient data for appraisal). Additional meta-exclusion motifs (e.g., missing test-set AUC/CI/SE, missing class counts, unclear validation split or potential leakage, unstated threshold basis, or absent calibration) are also annotated. Additional methodological details are available in Supplementary File S1.

Search results were exported (RIS/CSV) and imported into a unified library. Records were de-duplicated using reference-manager rules complemented by DOI and normalized-title matching. Screening proceeded in two stages (title/abstract and then full text) by two reviewers independently, with disagreements resolved by consensus. From 158 records identified, 39 were duplicates, leaving 119 for screening and full-text assessment. After applying eligibility criteria, 17 studies were included in the qualitative synthesis; of these, 6 reported complete quantitative information for meta-analysis (Table 1) [1,3,6,7,11,12].

A piloted extraction form captured bibliometrics; modality (imaging sub-type; non-imaging category); model type and feature set; validation design (k-fold/hold-out/external) and dataset split; test-set sample size and class counts (BD/MDD); AUC with 95% confidence interval or standard error; if thresholded, the decision threshold and its basis; calibration metrics (e.g., slope, intercept, and Brier score); and any explicit data leakage checks. When CIs were not reported but SEs or raw numbers permitted, missing quantities were derived using standard transformations. Studies lacking sufficient information for these derivations were retained for qualitative synthesis only.

Risk of bias and applicability were assessed using QUADAS-2 domains (participant selection, index test, reference standard, flow, and timing), adapted to diagnostic accuracy ML studies, with complementary PROBAST/AI considerations (data leakage, tuning on test data, repeat measurements, leakage via feature engineering, and handling of missingness). Domain judgments were categorized as low, high, or unclear risk by two assessors, with consensus resolution of disagreements. Studies judged to have a high risk of bias due to probable data leakage were prespecified for sensitivity analyses and potential exclusion from quantitative synthesis. Two reviewers rated each domain independently; disagreements were resolved by consensus.

The primary effect measure was the AUC evaluated on an independent test set (internal hold-out/k-fold test fold or external cohort). Where studies reported multiple models per modality, we prioritized the prespecified primary model; if none was explicitly designated, we selected the model with the highest test-set AUC within that modality for the main analysis and explored alternative models in sensitivity analyses. Threshold-based sensitivity and specificity were summarized narratively unless thresholds were sufficiently harmonized to permit pooled analyses. Calibration metrics were synthesized descriptively. AUCs were meta-analyzed on the logit scale and back-transformed to the original scale for reporting.

We performed random-effects meta-analyses of AUCs using the Hartung–Knapp adjustment with restricted maximum likelihood (REML) estimation of between-study variance (τ^2^). Standard errors were obtained from reported CIs or computed from AUCs and class counts when feasible, and generic inverse-variance weighting was applied. Heterogeneity was summarized with τ^2^ and I^2^, and 95% prediction intervals were reported when ≥3 studies were available. Given the small number of eligible studies, τ^2^ and subgroup/moderator inferences were interpreted cautiously, and emphasis was placed on effect size estimates with uncertainty (CIs and prediction intervals) rather than heterogeneity tests alone.

Our a priori subgroup analysis contrasted imaging versus non-imaging modalities. Sensitivity analyses excluded studies at high risk of bias or with potential data leakage (Xi (2023) [11] and Zeng (2023) [6] and compared internal versus external validation. Small-study effects and publication bias were explored with funnel plots and regression tests adapted to AUCs when the number of studies permitted, interpreted cautiously given known limitations for diagnostic metrics.

We summarized the certainty of evidence at the outcome level using a GRADE-adapted framework for diagnostic accuracy meta-analyses of AUC, considering risk of bias, inconsistency, indirectness, imprecision, and publication bias.

All analyses were performed using R, version 4.3.0 (R Core Team, R Foundation for Statistical Computing, Vienna, Austria; 2024). Data processing and coding were conducted in RStudio, version 2023.06.0 + 421 (Posit Software, PBC, Boston, MA, USA, 2023), an integrated development environment for R. Statistical significance was set at *p* < 0.05, two-tailed. An artificial intelligence–based language model (ChatGPT-5.2, OpenAI) was used solely for linguistic assistance during manuscript preparation, as detailed in the Acknowledgments.

## 3. Results

### 3.1. Summary Diagnostic Performance and Between-Study Heterogeneity

The random-effects meta-analysis including six studies yielded a pooled AUC of 0.837 (95% CI 0.754–0.895), indicating above-chance discrimination between bipolar disorder and unipolar major depression at first episode. Between-study heterogeneity was substantial (I^2^ = 86.5%, τ^2^ = 0.323), with a highly significant Q-test (Q = 36.96, df = 5, *p* < 0.0001), indicating that variability across studies reflects genuine differences rather than sampling error alone. The corresponding prediction interval on the logit-AUC (GEN) scale was wide, indicating that expected performance in a new study could plausibly range from modest to very high, underscoring strong contextual dependence. This degree of heterogeneity implies that model performance is not uniform across datasets and may be influenced by factors such as modality (imaging vs. non-imaging), sample size, validation design, or risk of data leakage. The significant Q-test further supports the presence of real between-study differences. Nonetheless, the pooled estimate remains statistically significant under the Hartung–Knapp adjustment, indicating that, despite heterogeneity, the included studies consistently show above-chance discriminatory performance. Because included studies span heterogeneous modalities and feature domains, the pooled estimate should be interpreted as a broad summary of ‘AI/ML for BD vs. MDD at first episode’ rather than a single clinically interchangeable model class. Threshold-based performance metrics (e.g., sensitivity and specificity) were reported in a subset of non-imaging studies (Zeng et al., 2023 [6]; Salvetat et al., 2024 [3]), including one study using a fixed probability threshold and external validation; however, no included study reported formal calibration metrics (e.g., calibration slope/intercept and Brier score) or decision curve analyses, precluding quantitative synthesis of clinical utility. The results are presented in Table 2.

**Figure 2:**
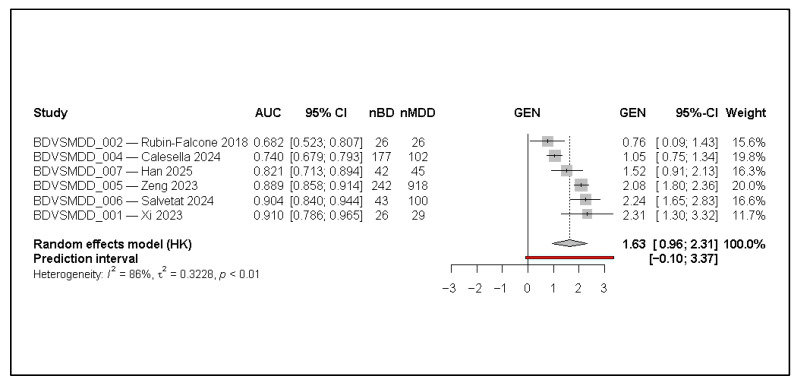
Forest plot of test-set AUCs for AI/ML models distinguishing bipolar disorder from unipolar major depressive disorder at first episode (random-effects model with Hartung–Knapp adjustment). AUC denotes the area under the receiver operating characteristic curve; 95% CI indicates the 95% confidence interval; nBD and nMDD represent the number of bipolar disorder and major depressive disorder cases in the evaluation set. Effect sizes are shown on the transformed logit-AUC (GEN) scale. Squares represent individual study estimates, with size proportional to inverse-variance weight; horizontal lines indicate 95% confidence intervals. The diamond represents the pooled random-effects estimate, and the red horizontal line denotes the 95% prediction interval [1,3,6,7,11,12].

The forest plot (Figure 2) displays the individual test-set AUCs from the six studies included in the quantitative synthesis. Reported AUCs range from 0.68 to 0.91, indicating substantial variability in diagnostic performance across datasets and model types. Studies with larger sample sizes (e.g., Zeng 2023 [6]) contribute greater precision, reflected in their narrower confidence intervals, but do not necessarily dominate the pooled estimate due to the random-effects weighting.

On the transformed effect scale (GEN), most studies cluster on the positive side of the axis, illustrating consistently above-chance discrimination. The pooled random-effects estimate (HK-adjusted) corresponds to a summary AUC of approximately 0.84, consistent with good diagnostic accuracy. The wide prediction interval (0.10–3.37 on the transformed scale) reflects substantial between-study variability and indicates that true study-level performance could differ meaningfully when models are applied to new populations or data sources.

Heterogeneity is high (I^2^ = 85.5%, τ^2^ = 0.32), confirming that the observed dispersion is not attributable to sampling error alone. This suggests that differences in data modality, sample composition (e.g., BD:MDD ratios), preprocessing strategies, and validation rigor materially influence model performance. Despite this heterogeneity, all included studies show AUCs above 0.65, and two exceed 0.90, indicating that AI/ML approaches can achieve robust discrimination in some settings.

### 3.2. Assessment of Publication Bias and Small-Study Effects

**Figure 3:**
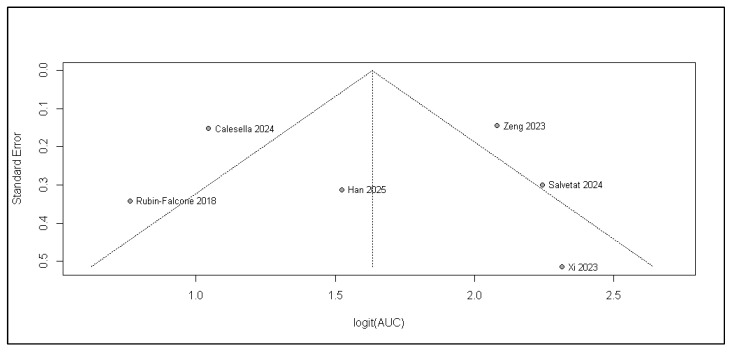
Funnel plot of standard error vs. logit (AUC) for included studies (k = 6) [1,3,6,7,11,12].

With only six studies, formal tests for funnel plot asymmetry are underpowered and cannot reliably detect publication bias or small-study effects. Although Egger’s regression (*p* = 0.92) and Begg’s rank test (*p* = 0.57) were not statistically significant, these results should be interpreted as insufficient evidence to assess bias rather than evidence of its absence. The results are presented in Figure 3.

### 3.3. Influence Analysis—Leave-One-Out Robustness of the Pooled Diagnostic Accuracy

Leave-one-out analyses showed that no single study disproportionately influenced the pooled point estimate, with recomputed GEN values ranging from 1.51 to 1.79 and overlapping confidence intervals. However, heterogeneity remained high across all iterations (I^2^ ≈ 75–89%), indicating that stability of the mean estimate does not imply uniform performance across settings.

**Figure 4:**
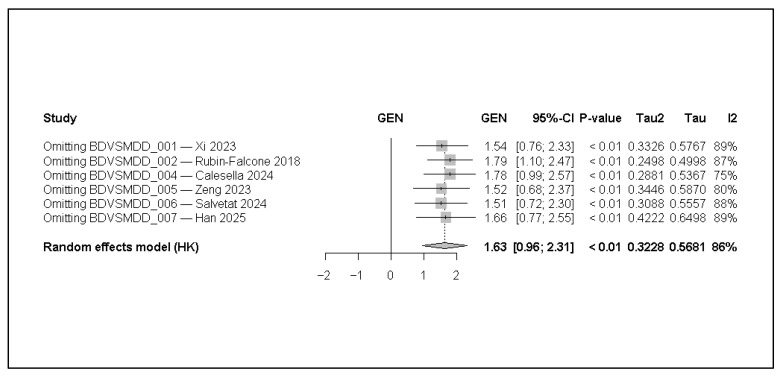
Leave-one-out sensitivity analysis using a random-effects model with Hartung–Knapp adjustment. The pooled effect size (GEN, logit-transformed AUC) was recomputed after omitting each study in turn (k = 6). Squares represent recalculated pooled estimates after exclusion of the indicated study, with horizontal lines indicating 95% confidence intervals. The diamond represents the overall pooled estimate from the full meta-analysis. Tau^2^ and Tau indicate between-study variance and its square root, respectively, while I^2^ represents the proportion of total variability attributable to between-study heterogeneity. Effect sizes are shown on the transformed logit-AUC (GEN) scale [1,3,6,7,11,12].

Overall, the LOO results demonstrate that the pooled point estimate was not driven by any single study (leave-one-out estimates were similar), but uncertainty remains substantial and prediction intervals indicate wide variability in expected performance across new settings. The wide prediction interval indicates that true performance in a future study could plausibly range from modest to very high, underscoring substantial contextual dependence. The results are presented in Figure 4.

### 3.4. Sensitivity to Study Quality—Pooled Accuracy After Excluding High-Risk-of-Leakage Studies

Excluding studies judged at high risk of data leakage reduced the evidence base to four studies. The pooled AUC was 0.800 (95% CI 0.682–0.882), closely aligned with the main analysis, though uncertainty increased, as reflected by wider confidence intervals.

**Figure 5:**
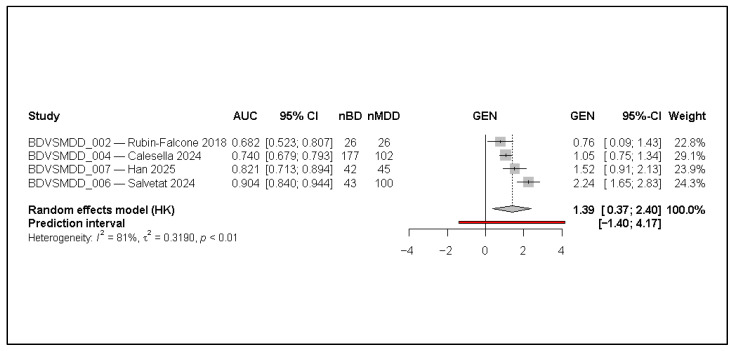
Random-effects meta-analysis using a Hartung–Knapp adjustment after excluding studies at high risk of data leakage (k = 4). AUC denotes the area under the receiver operating characteristic curve; 95% CI indicates the 95% confidence interval; nBD and nMDD represent the number of bipolar disorder and major depressive disorder cases in the evaluation set. Effect sizes are displayed on the transformed logit-AUC (GEN) scale. Squares represent individual study estimates, with size proportional to inverse-variance weight; horizontal lines indicate 95% confidence intervals. The diamond represents the pooled random-effects estimate, and the red horizontal line denotes the 95% prediction interval. Tau^2^ (τ^2^) indicates between-study variance, and I^2^ represents the proportion of total variability attributable to between-study heterogeneity [1,3,7,12].

Heterogeneity remained substantial (I^2^ = 80.7%), indicating that between-study variability is not primarily driven by leakage concerns alone. The results are presented in Figure 5.

### 3.5. Robustness Under Stricter Validation—Pooled Accuracy in High-Rigor Studies

**Figure 6:**
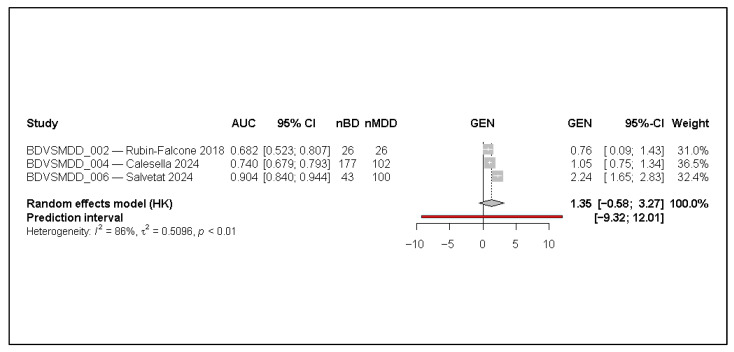
Random-effects meta-analysis using a Hartung–Knapp adjustment restricted to studies with high-rigor validation procedures (k = 3). AUC denotes the area under the receiver operating characteristic curve; 95% CI indicates the 95% confidence interval; nBD and nMDD represent the number of bipolar disorder and major depressive disorder cases in the evaluation set. Effect sizes are displayed on the transformed logit-AUC (GEN) scale. Squares represent individual study estimates, with size proportional to inverse-variance weight; horizontal lines indicate 95% confidence intervals. The diamond represents the pooled random-effects estimate, and the red horizontal line denotes the 95% prediction interval. Tau^2^ (τ^2^) indicates between-study variance, and I^2^ = 86% represents the proportion of total variability attributable to between-study heterogeneity [3,7,12].

Restricting the analysis to studies using stricter validation procedures (k = 3) yielded a pooled AUC of 0.794 (95% CI 0.616–0.902). Confidence and prediction intervals were wide, and heterogeneity remained high (I^2^ = 86.4%), emphasizing the limited precision achievable with few studies and reinforcing the exploratory nature of this subset analysis. The results are presented in Figure 6.

### 3.6. Comparative Performance by Modality—Imaging vs. Non-Imaging Subgroups

**Figure 7:**
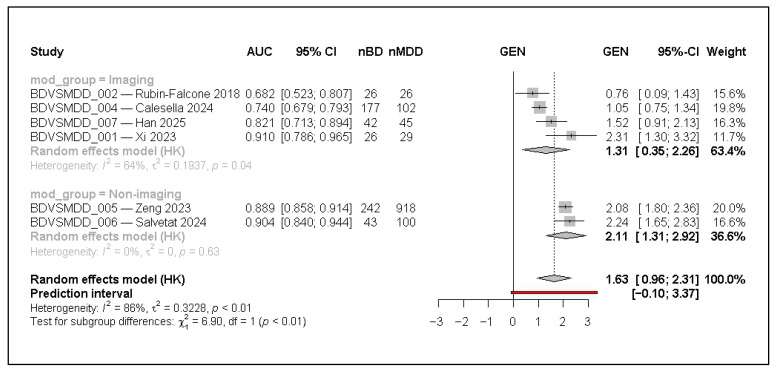
Subgroup meta-analysis using a random-effects model with Hartung–Knapp adjustment, comparing imaging (k = 4) and non-imaging (k = 2) studies. AUC denotes the area under the receiver operating characteristic curve; 95% CI indicates the 95% confidence interval; nBD and nMDD represent the number of bipolar disorder and major depressive disorder cases in the evaluation set. Effect sizes are displayed on the transformed logit-AUC (GEN) scale. Squares represent individual study estimates, with size proportional to inverse-variance weight; horizontal lines indicate 95% confidence intervals. Diamonds represent pooled random-effects estimates within each subgroup and overall. The red horizontal line denotes the 95% prediction interval for the overall model. Between-subgroup differences were assessed using a Q-test (Q = 6.90, *p* < 0.01); this subgroup analysis is exploratory and should be interpreted with caution given the small number of studies per subgroup [1,3,6,7,11,12].

In subgroup analyses, imaging studies (k = 4) yielded a pooled GEN of 1.31 (95% CI 0.35–2.26; I^2^ = 63.8%), corresponding to an AUC of approximately 0.79, whereas non-imaging studies (k = 2) showed a higher pooled GEN of 2.11 (95% CI 1.31–2.92; I^2^ = 0%), corresponding to an AUC of approximately 0.89–0.92. Although the test for subgroup differences was statistically significant (Q = 6.90, *p* = 0.009), this comparison is based on very small subgroup sizes—particularly for non-imaging models—and should therefore be interpreted as exploratory rather than confirmatory. Notably, the non-imaging studies tended to have larger evaluation samples than imaging studies, which may contribute to performance stability and could partially confound modality differences. The results are presented in Figure 7.

## 4. Discussion

Across six independent studies—two imaging task/sMRI studies (Rubin-Falcone et al., 2018 [7]; Xi et al., 2023 [11]), two additional imaging/structural pipelines (Calesella et al., 2024 [12]; Han et al., 2025 [1]), and two non-imaging approaches spanning clinical/EMR and blood biomarkers (Zeng et al., 2023 [6]; Salvetat et al., 2024 [3])—machine learning models showed above-chance discrimination between first-episode bipolar and unipolar major depression, with substantial between-study variability. Wide prediction intervals indicate that expected performance may vary greatly across settings. The pooled AUC (~0.84) remained stable after (i) removal of studies at high risk of data leakage, (ii) leave-one-out re-estimation, and (iii) restriction to stricter validation designs, indicating that the central result is robust to analytic choices and single-study influence

A key observation is the fact that non-imaging studies currently perform better in the available studies, potentially influenced by larger samples and lower site heterogeneity. Studies leveraging structured clinical features and/or blood-based signals (Zeng et al., 2023 [6]; Salvetat et al., 2024 [3]) achieved pooled AUCs around 0.88–0.90 with minimal heterogeneity, suggesting that this apparent consistency likely reflects the small number of studies rather than definitive uniform performance. In contrast, imaging pipelines (Rubin-Falcone et al., 2018 [7]; Xi et al., 2023 [11]; Calesella et al., 2024 [12]; Han et al., 2025 [1]) pooled lower (~0.79) and displayed wider dispersion, consistent with site effects, harmonization challenges, and smaller/less balanced samples typical of MRI research. Notably, task-fMRI using working memory load (Xi et al., 2023 [11]) performed well within its study but did not erase the overall modality gap at the meta-analytic level

Heterogeneity was high and persisted after excluding high-risk studies, indicating true between-study differences rather than artifacts of bias. Likely contributors include (i) case-mix and class imbalance (e.g., Zeng et al., 2023 [6] with large MDD cohorts vs. smaller BD cohorts), (ii) preprocessing and feature handling (e.g., fold-wise normalization vs. global scaling), (iii) validation design (nested cross-validation vs. open test splits), and (iv) measurement domain (blood/clinical vs. MRI). Importantly, the high I^2^ values observed do not invalidate the findings, but rather reflect genuine methodological and population-level variability that is common in AI-based diagnostic research, particularly across heterogeneous data modalities and clinical settings. Despite these differences, all included studies reported above-chance discrimination (AUCs ≥ ~0.68), underscoring the presence of this effect across diverse methodological approaches, despite substantial variability in performance (Rubin-Falcone et al., 2018 [7]; Xi et al., 2023 [11]; Zeng et al., 2023 [6]; Calesella et al., 2024 [12]; Salvetat et al., 2024 [3]; Han et al., 2025 [1])

QUADAS-2 flagged index-test concerns in several imaging pipelines due to potential data leakage or unclear fold-wise preprocessing, while reference-standard risk was low across studies. Excluding studies at high risk of leakage minimally altered the pooled point estimate, suggesting that leakage concerns did not solely drive the observed effect. Formal assessments of publication bias were underpowered given the small number of studies (k = 6) and should be interpreted as insufficient to detect small-study effects rather than evidence of their absence.

The meta-analytic signal supports pragmatic deployment pathways prioritizing non-imaging inputs—structured clinical/EHR variables and blood biomarkers—which are less costly, easier to standardize, and performed best (Zeng et al., 2023 [6]; Salvetat et al., 2024 [3]). Imaging may add value in specific contexts (e.g., task-fMRI paradigms capturing cognitive control; Xi et al., 2023 [11]), but current evidence suggests lower and less consistent gains at the point of first-episode differential diagnosis. These findings align with a step-wise diagnostic support paradigm: low-burden non-imaging models for triage, reserving imaging for unresolved cases or mechanistic clarification. From a clinical implementation perspective, such models are best conceptualized as adjunctive decision-support or triage tools, integrated within existing electronic health record systems to flag patients who may warrant closer longitudinal assessment, rather than as standalone diagnostic instruments. This positioning aligns with current regulatory and clinical expectations and mitigates risks associated with over-reliance on algorithmic classification at first presentation.

Strengths include protocol pre-registration on protocols.io, multi-database retrieval, duplicate screening/deduplication, quantitative synthesis on the logit-AUC scale, and a suite of robustness checks (leave-one-out, exclusion of high-risk studies, and a high-rigor subset). Limitations are inherent to the evidence base: small k, heterogeneity, limited calibration reporting (few studies provided slopes/Brier scores), and incomplete transparency about leakage controls in some pipelines. Several studies were cross-sectional or retrospective; prospective, site-level external validation remains sparse (an exception being the multicentric external validation in Salvetat et al., 2024 [3]). Because the quantitative synthesis included only six studies, estimates of between-study variance (τ^2^) and heterogeneity metrics (I^2^) are inherently uncertain; findings should be viewed as provisional and hypothesis-generating. Several imaging studies had small evaluation samples, which increases uncertainty and limits the generalizability of their reported AUCs. By excluding healthy control comparisons, this review addresses diagnostic differentiation between BD and MDD but cannot determine whether reported features represent disorder-specific signals or general markers of illness severity or distress. Consequently, findings should be interpreted as relative discrimination between two clinical populations rather than absolute disease specificity [3].

Priorities include (i) prospective, multi-site external validation with locked pipelines (Salvetat et al., 2024 [3] as a template), (ii) standardized, fold-wise preprocessing and reporting of leakage checks, (iii) calibration and clinical utility reporting (thresholds and decision curves), (iv) head-to-head comparisons of imaging and non-imaging models within the same first-episode cohorts to disentangle modality effects from sample size- and site-related confounding, and (v) exploration of multimodal fusion that preserves rigor (e.g., nested CV and site-level splits) to test whether carefully integrated imaging adds incremental value beyond clinical/biomarker features [3].

Taken together, the literature synthesized here (Rubin-Falcone et al., 2018 [7]; Xi et al., 2023 [11]; Zeng et al., 2023 [6]; Calesella et al., 2024 [12]; Salvetat et al., 2024 [3]; Han et al., 2025 [1]) supports the conclusion that machine learning can achieve above-chance discrimination between bipolar and unipolar depression at first episode. These results justify prospective clinical evaluation of non-imaging ML decision support for early diagnostic differentiation, while encouraging higher-rigor methodology and harmonization for imaging-based pipelines [1,3,6,7,11,12].

## 5. Conclusions

This meta-analysis demonstrates that machine learning models provide above-chance diagnostic discrimination between first-episode bipolar depression and unipolar major depression across the included studies. The pooled random-effects AUC was ~0.84, and estimates remained in the same accuracy range across all sensitivity checks—including exclusion of studies at high risk of data leakage, leave-one-out analyses, and restriction to studies using stricter validation—indicating that the overall estimate was not driven by any single study or analytic decision.

A modality effect emerged. Non-imaging models showed higher pooled point estimates than imaging models in the currently available literature, though this difference may be influenced by confounding factors such as larger sample sizes and reduced site/acquisition variability.

Between-study heterogeneity was high throughout (I^2^ ~ 80–90%) and remained high after excluding high-risk studies, implying that variability reflects genuine differences in datasets and modeling pipelines rather than a single influential study or obvious bias. The wide prediction intervals further indicate that expected performance in future studies may plausibly range from modest to very high, underscoring substantial contextual dependence.

Risk-of-bias assessment showed low concern for the reference standard but recurrent issues in the index-test domain related to ML pipeline reporting and potential leakage; nevertheless, the pooled accuracy was stable when high-risk studies were removed. Funnel plot symmetry and formal tests (Egger and Begg) provided no evidence of small-study or publication bias, acknowledging limited power with six studies.

Collectively, the evidence indicates that machine learning models can achieve above-chance discrimination between bipolar and unipolar depression at first episode, albeit with substantial variability across studies and settings. Non-imaging approaches showed higher pooled point estimates in the limited available literature, but evidence remains preliminary. These findings support the feasibility of ML-assisted diagnostic differentiation while emphasizing the need for cautious interpretation and prospective validation.

## Figures and Tables

**Table 1:** Characteristics of studies included in the meta-analysis.

First Author	Year	Journal	Modality	Model Type	Validation Type	N Evaluated BD	N Evaluated MDD
Xi [11]	2023	*Canadian Journal of Psychiatry*	task-fMRI n-back; graph metrics (degree centrality)	SVM (LIBSVM)	internal CV (details in Supplementary File S1)	26	29
Rubin-Falcone [7]	2018	*Journal of Affective Disorders*	sMRI VBM gray matter volume features	SVM (C-SVC, linear kernel, C = 1; LIBSVM)	internal_leave-two-out + external	26	26
Calesella [12]	2024	*Neuroscience Applied*	DTI metrics (FA/MD/AD/RD) ± VBM GM volumes (analyzed separately)	PCA + elastic net (logistic) with class weighting; pipeline inside nested CV	internal nested_kfold	177	102
Zeng [6]	2023	*npj Mental Health Research*	EHR/clinical (duration, age of onset) + hematology (CBC) + blood biochemistry panel (standard serum markers)	XGBoost (best); also LR, SVM, RF benchmarked; feature selection via ANOVA + SHAP	internal_holdout (repeated) + internal_kfold (within-train)	242	918
Salvetat [3]	2024	*Journal of Affective Disorders*	Blood biomarkers: RNA editing (A-to-I) signatures (transcriptome-derived editing sites)	Extra-Trees (ET)	external	43	100
Han [1]	2025	*Journal of Affective Disorders*	sMRI cortical folding/local gyrification index (LGI)	XGBoost	internal_kfold	42	45

**Table 2:** Pooled test-set AUC from random-effects meta-analysis (k = 6).

Statistic	Value
Pooled AUC (random-effects, REML + Hartung–Knapp)	0.837 (95% CI 0.754–0.895)
Number of studies (k)	6
Tau^2^ (between-study variance)	0.3228 (95% CI 0.0791–2.4639)
Tau	0.5681 (95% CI 0.2813–1.5697)
I^2^	86.5% (95% CI 72.7–93.3%)
Q statistic (df = 5)	36.9, *p* < 0.0001
Model type	Random-effects, inverse-variance, REML
Small-sample adjustment	Hartung–Knapp
Effect scale used in estimation	Logit-AUC (back-transformed for reporting)

## Data Availability

The datasets generated during the current study are available from the corresponding author on reasonable request.

## Supplementary Materials

The following supporting information can be downloaded at: https://www.mdpi.com/article/10.3390/jcm15020834/s1, File S1: The PRISMA checklist.

## Abbreviations

The following abbreviations are used in this manuscript:

AI	Artificial Intelligence
AUC	Area Under the Curve
BD	Bipolar Disorder
CI	Confidence Interval
CNN	Convolutional Neural Network
DTI	Diffusion Tensor Imaging
EEG	Electroencephalography
EMR	Electronic Medical Record
EHR	Electronic Health Record
GEN	Logit-AUC effect scale used for meta-analysis
HK	Hartung–Knapp
I^2^	Proportion of between-study variance
LOO	Leave-One-Out
LR	Logistic Regression
MDD	Major Depressive Disorder
ML	Machine Learning
MRI	Magnetic Resonance Imaging
NLP	Natural Language Processing
PI	Prediction Interval
PRISMA	Preferred Reporting Items for Systematic Reviews and Meta-Analyses
PROBAST	Prediction model Risk of Bias Assessment Tool
QUADAS-2	Quality Assessment of Diagnostic Accuracy Studies-2
REML	Restricted Maximum Likelihood
RF	Random Forest
ROC	Receiver Operating Characteristic
SE	Standard Error
SVM	Support Vector Machine
τ	tau (Standard deviation of between-study effects)
τ^2^	tau-squared (Between-study variance in random-effects meta-analysis)
